# Prevalence and Correlates of Vitamin D Deficiency in a Sample of 290 Inpatients With Mental Illness

**DOI:** 10.3389/fpsyt.2019.00167

**Published:** 2019-03-29

**Authors:** Alessandro Cuomo, Giuseppe Maina, Simone Bolognesi, Gianluca Rosso, Bruno Beccarini Crescenzi, Francesco Zanobini, Arianna Goracci, Edvige Facchi, Ettore Favaretto, Irene Baldini, Aurora Santucci, Andrea Fagiolini

**Affiliations:** ^1^Department of Molecular and Developmental Medicine, University of Siena, Siena, Italy; ^2^Department of Neuroscience, University of Torino, Turin, Italy; ^3^Azienda USL Toscana Sud Est, Arezzo, Italy; ^4^Ospedale di Bressanone, Bressanone, Italy

**Keywords:** mood, psychiatric, mental, inadequacy, deficiency, vitamin D, depression, bipolar

## Abstract

**Introduction:** Vitamin D inadequacy or deficiency (VDID) has been reported in a high percentage of otherwise healthy individuals. Factors that may contribute to the high prevalence of VDID in people with mental disorders include diet low in vitamin D, poor sunlight exposure, decrease in cutaneous vitamin D synthesis, intake of certain medications, poor mobility, excessive alcohol intake, and tobacco smoking. VDID has been correlated to a host of adverse conditions, including rickets, osteoporosis, osteomalacia, muscle diseases, depression, cognitive dysfunction, and even certain cancers.

**Objectives:** The purpose of this study was to report the prevalence and correlates of vitamin D inadequacy in a sample of 290 psychiatric patients admitted to inpatient or day hospital treatment at the University of Siena Medical Center.

**Methods:** We retrospectively evaluated the prevalence of VDID in 290 psychiatric inpatients' medical records during the year 2017 and evaluated the correlates of VDID in patients with mental illness.

**Results:** Two hundred and seventy two out of two hundred and ninety patients (94%) showed VDID. Physical activity and regular diet were positively correlated with vitamin D levels whereas age, tobacco smoking, PTH, alkaline phosphatase levels were negatively correlated. Statistically significant differences were found among smokers and non-smokers in all study groups.

**Conclusions:** VDID was highly prevalent in our sample. In addition to vitamin D supplementation, psychosocial intervention able to promote and help sustain physical activity, appropriate diet, quitting smoking and sensible sun exposure to prevent and treat VDID in patients with mental health should be implemented, tested, and introduced in our clinical practice.

## Introduction

Vitamin D (vit D), also known as sunshine vitamin is an essential component for a number of physiological functions such as muscle performance, bone metabolism, calcium and phosphorus omeosthasis and immunity. Vit D may be produced via sun exposure or obtained from food (1). According to Clinical Practice Guidelines from the US Endocrine Society, vitamin D deficiency is defined as serum 25-OH D <50 nmol/l (below 20 ng/ml). Vit D insufficiency is defined as serum levels of 25-OH D ranging between 50 and 75 nmol/l (21–29 ng/ml) (2, 3). Vit D deficiency is a global health issue affecting individuals of all ages (4). There are number of factors causing deficiency, with lack of sunlight exposure and poor dietary intake being the most important (5). The mean prevalence of Vit D deficiency in US population has been reported to be 37.5% (6). It is associated with various metabolic, neoplastic and immunological disorders such as atherosclerosis, diabetes mellitus and colon cancer (2, 7).

Vit D also plays an important role in mental health and cognitive functions (8, 9). Vit D receptors are present in various parts of the brain, including the amygdala, which is associated with the regulation of emotions and behavior (10). Vit D regulates intra and extracellular calcium concentration in neurons (11). Researchers found that patients with low vitamin D levels suffer from mood disorders (12, 13). In addition, vit D deficiency has been associated with neuropsychiatric conditions such as Parkinson's disease, schizophreniform disorder, multiple sclerosis (MS), Alzheimer's disease and autism spectrum disorders (14–16). Other studies have found significant relationships between deficiency of vitamin D and depressive symptoms or cognitive impairment (17, 18).

The main reasons for vitamin D deficiency in patients with psychiatric disease are low exposure to sunlight and poor diet (19). Anticonvulsant therapy may play a role as well (20). Although it is not a common practice to screen psychiatric patients for vitamin D deficiency, evidence suggests that vit D levels should be included in routine screening. For example, a study conducted in UK found that 100% psychiatric male inpatients were vitamin D deficient during hospital treatment (21). In another study conducted in the same country, researchers found that 83% of hospitalized inpatients were Vitamin D deficient (22).

The purpose of the study was to report the prevalence and correlates of vit D deficiency in a sample of 290 psychiatric patients admitted to inpatient or day hospital treatment at the University of Siena Medical Center.

## Methods

We retrospectively evaluated the medical records of 290 patients admitted to inpatient or day hospital treatment from January to December 2017.

The following information was extracted from the clinical charts: socio-demographic variables (age, sex, date, and place of birth); main psychiatric diagnosis; vitamin D serum levels and other parameters of the phosphocalcic metabolism (PTH, alkaline phosphatase, calcium, magnesium, and phosphorus); prolactin serum levels; body weight, height and body mass index (BMI); regular diet (as expressed by at least two meals per day); regular physical activity (as expressed by aerobic activity >20 min at least 2 days per week); smoking tobacco; medical comorbidities. All data above was collected retrospectively and no exam was specifically conducted for the purposes of this study. For the reasons above, the study was exempt from informed consent. The study was approved by the Regione Toscana - Area Vasta SudEst Ethical Committee Board.

We excluded confounding conditions that may contribute to low vitamin D levels such as malnutrition (eating disorders and/or wasting syndrome due to medical reasons) or malabsorption (celiac disease, inflammatory bowel disease, exocrine pancreatic insufficiency from cystic fibrosis and/or short bowel syndrome). Subjects with metabolic bone diseases (such as osteoporosis, rickets, osteomalacia, osteopetrosis, Paget disease of bone, and fibrous dysplasia) and pathological hyperprolactinemia not drug induced (such as pituitary micro- and macroadenomas, prolactinoma, chronic renal failure, and patients on hemodialysis) were also excluded.

Descriptive statistics were used to summarize the participant characteristics. Participants were then divided into three groups based on their vit d Levels: (1) vit D deficiency (vit D levels <10 ng/ml); (2) vit D insufficiency (vit D levels = 10–29.99 ng/ml); (3) vit D adequacy (vit D levels greater ≥30 ng/ml). The three groups were compared with chi-square tests on categorical variables such as gender, physical activity, smoke, diagnosis (bipolar disorder vs. other disorders), season (other seasons vs. summer) and drug treatment, and with Kruskal-Wallis test on quantitative variables (age, BMI, PRL, Magnesium, Calcium, Phosphorus, Alkaline phosphatase, and PTH) because they showed a non-normal distribution, as assessed using Shapiro-Wilks test. The significance level was set at *p* < 0.05. Variables that differed significantly among the three groups of vitamin D levels were entered as independent variables in a logistic regression model with a forward stepwise procedure. In this model, vitamin D was used a dependent variable, coded as deficiency vs. no deficiency. Statistical analyses were performed using SPSS for Windows version 22.0 and PRISM GraphPad.

## Results

The sample consisted of 290 patients, of which 127 were males (43.8%) and 163 females (56.2%). The mean age was 47.8 years. The majority of patients had a diagnosis of mood disorders (*n* = 251, 86.6%): among these 243 (83.8%) had a diagnosis of bipolar disorder and 8 (2.8%) of major depressive disorder; other diagnoses were schizophrenia or psychosis (*n* = 8; 2.8%), post-traumatic stress disorder (*n* = 17; 5.9%), other mental disorders (anxiety disorders, neurodevelopmental disorder) (*n* = 14; 4.8). The mean values for the anthropometric parameters of the sample were: weight 75 kg, height 169 centimeters, BMI 26.2 kg/m^2^. About three quarters of patients (219/75.5%) followed a regular diet, 147 (50.7%) were smokers, and 75 (25.9%) reported to practice regular physical activity.

Ninety-four percent (*n* = 272) of the 290 study subjects showed vit D levels below the normal range (vit D ≥30 ng/ml). Specifically, 31% met criteria for vit D deficiency (vit D levels <10 ng/ml), and 63% had vit D insufficiency (vit D levels = 10−30 ng/ml). The mean vitamin D levels was 15.3 ng/ml (±7.7 ng/ml). Not surprisingly, vit D levels were higher in samples collected in the months of July and August. Vit D levels were inversely proportional to age (*p* < 0.001). No significant relationship between vitamin D levels and gender was found (*p* = 0.481). Physical activity (*p* < 0.001) and regular diet (*p* < 0.01) were found to be positively and significantly related to vitamin D levels. Tobacco smoking had a negative association with vitamin D levels (*p* < 0.001). No statistically significant correlations were found between vitamin D levels and calcium, magnesium, phosphorus and prolactin. However, PTH (*p* < 0.001) and alkaline phosphatase (*p* < 0.001) were inversely and significantly related to vitamin D levels. Psychiatric medications were unrelated with vitamin D levels (*p* = 0.935). Demographic and clinical features of the study sample divided in three groups based on vitamin D levels (deficiency: <10 ng/ml; insufficiency: 10–30 ng/ml; adequacy: ≥30 ng/ml) are presented in Table 1.

**Table 1:** Demographic and clinical characteristics of study participants according to vit D levels.

**Variables**	**Deficiency <10 ng/ml**	**Insufficiency 10–30 ng/ml**	**Adequacy ≥30 ng/ml**	***P*-value**
*N*	89	183	18	**–**
Males, *n* (%)	39 (43.8)	82 (44.8)	6 (33.3)	χ^2^ = 0.87677, df = 2, *p* = 0.645
Physical activity, *n* (%)	10 (11.2)	49 (26.8)	16 (88.9)	**FET**, ***p*** **<** **0.0001**
Smoke, *n* (%)	68 (76.4)	*79 (43.2)*	0 (0.0)	**χ**^**2**^ ***=46.189, df=2, p≤0.0001***
Age (Median)	54.4	45.2	48.1	**KW** **=** **13.284, df** **=** **2**, ***p*** **<** **0.01**
BMI (Median)	26.2	26.1	26.9	KW = 0.295, df = 2, *p* = 0.863
Drug treatment, *n* (%)	81 (91.0)	161 (88.0)	15 (83.3)	FET, *p* = 0.524
PRL (Median)	275.3	272.5	318.2	KW = 0.1223, df = 2, *p* = 0.941
Magnesium (Median)	2.1	2.0	2.1	KW = 3.476, df = 2, *p* = 0.176
Calcium (Median)	9.4	9.5	9.5	KW = 1.3197 df = 2, *p* = 0.517
Phosphorus (Median)	3.2	3.2	3.3	KW = 0.47053, df = 2, *p* = 0.790
Alkaline phosphatase (Median)	68	59	61	**KW** **=** **12.585, df** **=** **2**, ***p*** **<** **0.01**
PTH (Median)	37.0	28.0	25.5	**KW** **=** **18.558, df** **=** **2**, ***p*** **<** **0.0001**

*BMI, Body Mass Index; df, degrees of freedom; FET, Fisher Exact Test; KW, Kruskal-Wallis; N, Number; ng/mL, Nanograms Per Milliliter; PRL, Prolactin; PTH, parathyroid hormone; χ^2^, chi-square. Bold values statistically significant*.

In the multiple logistic regression model, smoking was the strongest predictor of vit D deficiency (OR = 5.033, 95% CI 2.684–9.435) after adjusting for the effect of season. Older age and higher PTH values were also significantly associated with an increased likelihood of vit D deficiency, while physical activity was associated with a lower likelihood of vit D deficiency (the *p*-value was borderline significant, *p* = 0.053).

## Discussion

We found a very high prevalence of VDID, with only 18 (6%) out of 290 patients with mental illness (mostly bipolar disorder) showing adequate levels of vitamin D. Our results are in line with similar studies (20, 21) and indicate that VDID is much greater in patients with mental illness than in the general population (6). A recent study evaluated vitamin D serum levels in a representative sample of 55,844 European individuals, and found that 13.0% had serum vit D concentrations of less 30 nmol/L (23). The authors concluded that these rates are concerning and call for action from a public health perspective.

Our findings highlight a much higher degree of concern in individuals with mental illness and point to the need of routinely screening for vitamin D deficiency, as a part of the standard assessment of patients with mental illness. However, given that almost all patients resulted deficient in vitamin D, the possibility to offer vitamin D supplementation to every patient with mental illness could be considered, as a cost-effective alternative to general screening. In addition to other benefits, vit D supplementation may improve the outcomes of illnesses like depression (24).

It is well-known that the main factors behind vitamin D deficiencies include the lack of sunlight exposure, poor dietary intake, smoking, and lack of physical exercise (5). Several studies have shown that a high number of patients with bipolar illness smoke tobacco, run a sedentary lifestyle, and have unhealthy dietary habits (25–28), similarly to what has been described in patients suffering from schizophrenia (27). Consistently with other studies in non-psychiatric patients [for instance; (29)], smokers were more likely to show VDID than non-smokers, despite the fact that outdoor smoking likely increased the sun light exposure. Of interest, in the multiple logistic regression model, smoking was the strongest predictor of vit D deficiency. This may be due to the fact that the negative influence of smoking on vitamin D levels is higher than the positive influence of a few minutes of sun exposure wen people smoke outdoors. Also, this could be due to the fact that our patients smoked primarily indoor. In fact, in our inpatient unit, people are allowed to smoke in a designated smoking area consisting of a fenced and shaded balcony, with no direct sunlight exposure. It is likely that our patients smoked indoors before being admitted as well, given that a comprehensive smoking ban (e.g., no smoking policies in apartment buildings) is not widely adopted/enforced in Italy. Although we did not find a significant relationship between VDID and specific medications, we cannot rule out this possibility, because polytherapy was common. For instance, the effect of antiepileptic drugs on vitamin D levels has been widely demonstrated.

Our study has several other limitations, including the retrospective design of the study, the evaluation of vit D levels via blood samples collected in different seasons of the year, and the heterogeneity of the sample in terms of psychiatric diagnosis and socio-demographic features.

## Conclusions

VDID is highly prevalent in patients with mental illness and is significantly influenced by lifestyle factors, such as diet, physical inactivity and tobacco smoking (this last with a strong and negative influence). As we already demonstrated (29) for other conditions affecting patients with mood disorders, the development and testing of standardized psychosocial healthy lifestyle interventions is warranted, as a key tool to promote and sustain physical and mental well-being of our patients.

## Data Availability

The datasets generated for this study are available on request to the corresponding author.

## Author Contributions

All authors listed have made a substantial, direct and intellectual contribution to the work, and approved it for publication.

### Conflict of Interest Statement

The authors declare that the research was conducted in the absence of any commercial or financial relationships that could be construed as a potential conflict of interest.
